# Interpretation of infant facial expression in the context of maternal postnatal depression

**DOI:** 10.1016/j.infbeh.2010.03.002

**Published:** 2010-06

**Authors:** Alan Stein, Adriane Arteche, Annukka Lehtonen, Michelle Craske, Allison Harvey, Nicholas Counsell, Lynne Murray

**Affiliations:** a University of Oxford, Oxford, United Kingdom; b University of California, Los Angeles, CA, United States of America; c University of California, Berkeley, CA, United States of America; d University of Reading, Reading, United Kingdom

**Keywords:** Postnatal depression, Cognitive bias, Baby face, Mother–child interaction

## Abstract

Postnatal maternal depression is associated with difficulties in maternal responsiveness. As most signals arising from the infant come from facial expressions one possible explanation for these difficulties is that mothers with postnatal depression are differentially affected by particular infant facial expressions. Thus, this study investigates the effects of postnatal depression on mothers’ perceptions of infant facial expressions. Participants (15 controls, 15 depressed and 15 anxious mothers) were asked to rate a number of infant facial expressions, ranging from very positive to very negative. Each face was shown twice, for a short and for a longer period of time in random order. Results revealed that mothers used more extreme ratings when shown the infant faces (i.e. more negative or more positive) for a longer period of time. Mothers suffering from postnatal depression were more likely to rate negative infant faces shown for a longer period more negatively than controls. The differences were specific to depression rather than an effect of general postnatal psychopathology—as no differences were observed between anxious mothers and controls. There were no other significant differences in maternal ratings of infant faces showed for short periods or for positive or neutral valence faces of either length. The findings that mothers with postnatal depression rate negative infant faces more negatively indicate that appraisal bias might underlie some of the difficulties that these mothers have in responding to their own infants signals.

## 1 Introduction

The early post partum period is vital for the development of the early mother–child responsivity and the mother–child relationship. There is good evidence that maternal postnatal depression is associated with a range of difficulties in maternal responsiveness and subsequently the child's development (Murray & Cooper, 2003). In particular, mothers with postnatal depression have difficulty in responding contingently to infant signals (Murray, Kempton, Woolgar, & Hooper, 1993; Stanley, Murray, & Stein, 2004). However, to date, the process underlying this difficulty has not been fully elucidated. As most signals arising from the infant come from facial expressions (Papousek & Papousek, 1977), one possibility is that mothers with postnatal depression are differentially sensitive to certain infant facial expressions.

While not specifically focussed on facial expression of emotion, the work of Field, Morrow, and Adlestein (1993) has shown, for example, that depressed mothers are less likely to agree with independent researchers’ ratings of infant negativity and more likely to give higher negativity scores than were non-depressed control mothers, suggesting differences at the level of perception. Murray, Fiori-Cowley, Hooper, and Cooper (1996) also found that depressed mothers responded more to infant expressions of negative affect than control mothers and, moreover, that the nature of their responsiveness differed. While such naturalistic observational findings are important, the factors influencing this maternal behaviour and in particular the nature of the mother's processing of infant facial expressions of emotion requires further investigation.

A further question concerns the specificity of the effects of depression. Thus, while the adverse effects of depression on maternal sensitivity to the infant have been well established, far less is known about the effects of other psychiatric disorders commonly occurring after childbirth such as anxiety, and the findings have been inconsistent. In one study mothers with GAD were *not* found to be less sensitive to their infants during face-to face interactions than non-anxious control group mothers (Murray, Cooper, Creswell, Schofield, & Scak, 2007), although another (Nicol-Harper, Harvey, & Stein, 2007) showed that mothers with elevated anxiety symptoms were less responsive in interactions with infants.

Evidence concerning cognitive processing of emotional expressions in adults from experimental studies provides a number of hypotheses that can be applied to infant studies. For example, it is well established that depression is characterized by cognitive attention biases favouring negative information, especially when self-related (Mathews & MacLeod, 2005), as well as by a deficit in processing positive affect that is associated with negative appraisal of ambiguous emotional information (Beevers, Wells, Ellis, & Fischer, 2009). These include distorted interpretation of interpersonal information such as others’ emotions and facial expressions (Bouhuys, Bloem, & Groothuis, 1995).

Notably, and consistent with the observational infant work described above, studies on appraisal of facial expressions have found that depressed individuals display a bias to interpret neutral or ambiguous facial information as negative (Hale, 1998; Mogg, Millar, & Bradley, 2000). Generally, depressed people also tend to valence happy faces as neutral, neutral faces as sad (Gur et al., 1992) and sad faces as very sad (George et al., 1998). In addition, research on attentional bias has shown that depressed people selectively attend to specific emotional cues in facial expressions (i.e. sad emotions) (Gotlib, Krasnoperova, Joorman, & Yue, 2004), although this bias has not been found in all studies (e.g., Bouhuys, Geerts, Mersch, & Jenner, 1996; Mikhailova, Vladimirova, Iznak, Tsusulkovskaya, & Sushko, 1998). Importantly, studies have found that both appraisal and attentional biases are not always observed. For instance, appraisal biases seem to be specific to ambiguous cues (i.e. prompted by complex social cues such as the ones observed in real life; Beevers et al., 2009) and attentional biases, such as engagement bias towards negative faces, are likely to be found only when the emotional valence of the expression is consciously perceived (Frewen & Dozois, 2005); for this reason, length of stimulus presentation is thought to play a role in the research findings to date (Donaldson, Lam, & Mathews, 2007).

As noted above, most of the studies on cognitive biases in depression and appraisal of facial expressions have been conducted using adults’ faces and little is known about biases in interpreting infant facial expressions. This is important because infant faces have a different configuration from those of adults (Lorenz, 1971) including a relatively large head, predominance of the brain capsule, large and low lying eyes and bulging cheek region, which is thought to be important for eliciting parental responses (Lorenz, 1971). Parents and, in particular, mothers’ interpretation of infants’ emotional expressions are crucial to contingent responsiveness which in turn predicts later child development (Yarrow et al., 1984). Because of the evidence that mothers with postnatal depression have difficulties in responding appropriately to their infants, and in particular respond negatively to their infants’ own negativity, this study aims to examine whether mothers with postnatal depression rate infant facial expressions more negatively than control mothers. Furthermore, in order to explore whether any biases found are specific to depression, and not to more general postnatal psychiatric disturbance, comparison is made with a group of mothers suffering from anxiety. Although cognitive biases are also observed in anxiety disorders, those are by and large characterised by specific attention biases towards threatening rather than negative information (Mogg et al., 2000). In order to examine these questions we used images from a range of different infants, each showing a spectrum of expressions. Our main hypotheses are: (H1) Depressed participants will display a negative appraisal bias and will therefore valence neutral and negative faces as more negative than control participants. (H2) The negative appraisal bias will be specific to depression, and not a result of general postpartum disorder; thus, anxious postnatal mothers will not show appraisal bias when compared to controls. (H3) The negative appraisal bias will be more prominent for stimuli shown for longer presentations.

## 2 Method

### 2.1 Participants

Participants were part of the Oxford Parent Project (OPP). Mothers were drawn from a community sample mainly recruited from the postnatal wards of the John Radcliffe Hospital. Women who met inclusion criteria (i.e. 18 years or over, sufficient level of English, living within a 35 miles radius of Oxford, no medical complications, principal infants’ caretaker, over 35 weeks gestation, baby with birth weight not below 2000 g and with no life threatening complication) and who agreed to take part in the study, were sent screening questionnaires approximately nine weeks after giving birth. Screening questionnaires included the Edinburgh Postnatal Depression Scale (EPDS; Cox, Holden, & Sagovsky, 1987) and the Generalised Anxiety Disorder Questionnaire (GAD-Q) (Newman et al., 2002). All mothers who scored above the threshold on either the EPDS (>12) or GAD-Q (>5.70), and a random sample of women who scored below the cut-off on both questionnaires were interviewed at home at 3 months using the Structured Clinical Interview for DSM Diagnosis (SCID; First, Spitzer, Gibbon, & Williams, 1996, research version). In addition to the SCID, the interviewer also rated the impact of the disorder upon the mother's life using the Clinician's Severity Rating (CSR; DiNardo, O’Brien, Barlow, Waddell, & Blanchard, 1983). Mothers who fulfilled the clinical criteria for depression or Generalised Anxiety Disorder (GAD) in the diagnostic interview were considered eligible for the study. In addition to scoring below threshold in the screening questionnaires, control mothers were included in the study if they met criteria for no present or past psychiatric disorder. During the 3 months home visit mothers completed the face rating task after the SCID interview. The first 15 women who received a principal diagnosis for a depressive disorder (DEP) were recruited into the current study and were matched with 15 controls and 15 mothers with GAD. Based on the diagnostic interview at 3 months, of the 30 case mothers recruited into this study four were comorbid for depression and anxiety. Of these, three had a principal diagnosis of depression and one had a principal GAD diagnosis. Mothers were matched for infant birth order and educational background. Mean mother age was 33 years (SD = 5), infants’ mean birthweight was 3.6 kg (SD = 0.55) and infants’ mean age at the 3 months’ visit was 3.60 months (SD = 0.85). There were no differences across groups on these two variables (see Table 1 for sample details) and these characteristics are representative of the full study sample. Approval for the study was obtained from the Oxfordshire Research Ethics Committee (reference number 003.004).

### 2.2 Screening questionnaires

1. *Edinburgh Postnatal Depression Scale (EPDS)*: The EPDS consists of 10 questions specifically designed for screening postnatal depression avoiding the confounds between depression symptoms and complaints typical in early motherhood. The questionnaire has a sensitivity of 86% and a specificity of 78% and has shown acceptable internal consistency (Cronbach's alpha *α* = 0.87; Cox et al., 1987).
2. *Generalised Anxiety Disorder Questionnaire (GAD-Q)*: The GAD-Q is a validated self-report diagnostic questionnaire on GAD, as defined by the DSM-IV. It includes questions about the nature of worry, the topics of worry, the somatic symptoms of GAD, and the amount of impairment that worry is causing in the respondent's life. The specificity and sensitivity has been reported to be over 80% and the internal consistency has been demonstrated to be good (*α* = 0.84) (Newman et al., 2002).
3. *Structured Clinical Interview for DSM Diagnosis (SCID)*: The SCID is a semi-structured diagnostic interview for assessing the DSM (American Psychiatric Association 1994) Axis I and Axis II disorders. Evidence of reliability and validity has been extensively reported (First et al., 1996).
4. *Clinician's Severity Rating (CSR)*: This is a 0–8 scale indicating the level of distress and/or impairment caused by the disorder; a score of four or greater indicates clinical severity. Adequate interrater reliability has been demonstrated for CSRs with kappa coefficients ranging from *k* = .67 to *k* = .86 for the various anxiety disorders (DiNardo et al., 1983).

### 2.3 Stimuli and procedure

The task was presented on a 12″ laptop monitor. Fifty baby faces were used in the study, ten for each of five target emotions (positive, muted positive, neutral, muted negative, negative). Images were drawn from a database of digital photographs of 27 infants who were filmed at home (see Kringelbach et al., 2008 for details). Faces were shown as greyscale images and matched for size and luminosity. Participants rated each facial expression using a likert-scale ranging from −9 (very negative) to +9 (very positive). Keyboard buttons were labelled accordingly and only designated response keys were registered by the computer. Participants were told that we were interested in the communication between parents and children and that since interpreting facial expressions is part of that communication they were going to be asked to rate babies’ facial expressions. After six practice trials, each face was shown twice in randomized orders, for a short (100 ms) and for a long (2000 ms) duration in order to investigate effects of length of exposure (Donaldson et al., 2007; Frewen & Dozois, 2005). Average scores were computed for each facial expression in each length condition.

## 3 Results

### 3.1 Overview of data analysis

Distributions of average appraisal score in each facial expression and in each length were examined and all met criteria for normality. Multivariate analyses of variance (MANOVAs) followed by inspection of individual analysis of variance (ANOVA) were used to investigate H1 and H2 in our original three recruitment groups (control, DEP, GAD). In a second step, given our particular interest in the specificity of depression and because an overlap of high anxiety and depression symptoms could undermine the differences between our two index groups, we examined the differences between GAD and DEP without the four comorbids of our sample. Next, we examined the effect of length of presentation (H3) via repeated measures analysis of variance. Finally, because experience of motherhood is likely to affect the appraisal of babies’ faces we investigated effects of parity on participants’ ratings of baby facial expressions using a series of MANOVAs.

#### 3.1.1 Participant emotional status effect

MANOVAs followed by examination of individual ANOVAs were performed to investigate Group by Stimulus interaction effects for short and long presentations separately (see Table 2). In long presentations, the main Group effect was not significant (*F*(10, 78) = 1.57, *ns*), however, a significant Group effect was observed for negative faces. Bonferroni-adjusted pairwise comparisons revealed that the DEP group rated negative faces as more negative than the control group (*p* = 0.028). There were no significant differences between control and GAD groups. Although in muted negative faces the DEP group also tended to rate faces more negatively than the control group, the difference did not reach statistical significance (*p* = 0.074). In short presentations, the main effect of group was not significant (*F*(10, 78) = 1.35, *ns*) neither were any of the individual ANOVAs for each Stimulus.

When the four women who were comorbid for depression and anxiety were removed and the two index groups were compared, there was no main group effect in long presentations (*F*(5, 20) = 0.78, *ns*). Likewise, there was no evidence of group differences in any of the specific long presentation conditions examined. A similar pattern was observed for negative faces in long presentations, however the previously observed group difference was no longer significant (*F*(1, 24) = 3.50, *p* = 0.073). In short presentations there was no significant main group effect (*F*(5, 20) = 0.49, *ns*). Likewise, inspection of specific conditions revealed no differences between GAD and DEP in any specific condition.

#### 3.1.2 Length of presentation effect

A 2 (Duration) by 3 (Group) repeated measures analysis of variance was performed for each Stimulus separately. Results revealed no evidence of length effect in neutral faces (*F*(1, 42) = 1.30, *p* = 0.27, *η*^2^ = .03). However, participants tended to give more extreme ratings when either positive or negative baby faces were presented for a longer time. Although this pattern was observed for faces representing both negative (negative *F*(1, 42) = 77.08, *p* < 0.0001, *η*^2^ = .65; muted negative *F*(1, 42) = 29.86, *p* < 0.0001, *η*^2^ = .42); and positive emotions (positive *F*(1, 42) = 8.81, *p* < 0.001, *η*^2^ = .17; muted positive *F*(1, 42) = 6.62, *p* = 0.01, *η*^2^ = .14). The effect sizes were stronger in the former condition (i.e. negative and muted negative). The above pattern did not vary as a function of Group (all *p*'s > 0.20).

#### 3.1.3 Parity

Parity effects were examined in all conditions. MANOVAs followed by inspection of ANOVAs of interest revealed that primiparous(_p_) mothers tended to perceive positive baby faces as more positive than non-primiparous(_np_) mothers. This finding was observed in positive faces for both short [*F*(1, 42) = 5.91, *p* = 0.019, *η*^2^ = .12; *M*_p_ = 6.8 (SD = 1.23) *vs. M*_np_ = 5.8 (SD = 1.21)] and long presentations [*F*(1, 42) = 4.39, *p* = 0.04, *η*^2^ = .09; *M*_p_ = 7.1 (SD = 1.16) *vs. M*_np_ = 6.3 (SD = 1.08)]. There were no significant overall parity effects in either short or long presentations, nor significant specific parity effects in any other condition.

## 4 Discussion

The aim of this study was to examine whether mothers with postnatal depression showed a systematic bias in their interpretation of infant facial expressions, and whether any biases found were specific to postnatal depression or common to both depression and anxiety. One further issue, which had not previously been studied in baby faces, was whether the length of viewing had any effect on the outcome. We found that all Groups rated baby faces as more negative or more positive when shown for a longer period than a shorter period of time. This is consistent with previous research on adults’ stimulus (Donaldson et al., 2007; Frewen & Dozois, 2005) which suggests that length of exposure to the emotional face stimuli affects ones’ judgment of the valence of the expression. More importantly we found that negative faces seem to be particularly susceptible to the effects of timing, with more negative interpretations being elicited by longer exposures. This suggests that conscious perceptions of the sad emotion seem to be associated with the appraisal bias.

Mothers in the depression group rated infant negative faces as more negative compared to the controls over the longer exposure condition. This was not evident in the shorter exposure period, and there were no differences for positive faces. These data support previous research concerning negativity bias when rating faces with a negative valence in the context of depression (Bouhuys et al., 1995; George et al., 1998), as well as previous research on the responses of depressed mothers to infant negative emotion (Field et al., 1993; Murray et al., 1996). It is also consistent with the findings of Weinberg, Olson, Beeghly, and Tronick (2006) that differences in interactions between depressed and well mothers emerged only under conditions of interaction stress. Notably, the fact that the findings of the current study were observed in mothers’ responses to infant faces suggests that similar biases in cognitive processes might underlie some of the difficulties observed in early relationships between infants and postpartum depressed mothers. Our results support observational evidence that when mothers are exposed to prolonged negative expressions on infant faces, they are more likely to interpret them more negatively, and this was specific to depression. Conceivably, this bias may affect the nature of their responsiveness to infants. It should also be noted that these were not the mothers’ own infants but a range of infant faces not known to them. It might be expected that mothers, especially depressed mothers, would be more affected by their own infants’ facial expression—although as yet there is no empirical evidence for this. First time mothers were more likely to rate positive faces more positively. While again no similar studies have been conducted, it might be speculated that this might enable new mothers to engage with their infants.

Notably there was scarce evidence for the effects of maternal GAD on maternal response to infant facial expression. This is consistent with the findings of observations of such mothers’ behavioural sensitivity to their infants being similar to controls (Murray et al., 2007).

The strengths of the study included that this was a tightly controlled experimental examination to explore the mothers’ responses to a range of infants’ facial expressions; the mothers were well matched and the study was designed to ascertain the specific effect of depression by including a group of postnatally anxious mothers for comparison. The study had a number of limitations: the sample was relatively small and we did not include facial expressions of the mothers’ own infants.

In conclusion this study found that mothers suffering from postnatal depression were more likely to rate infant faces more negatively than controls and that this was specific to depression rather than a general effect of postnatal psychopathology. Further research needs to investigate whether such biases in interpretation of infant faces translates into a mother's behavioural responsivity to her infant.

## Figures and Tables

**Table 1:** Demographics and screening characteristics by group.

	Control	Anxiety	Depression	Statistics
Mother age in years	32.7	33.3	33.6	*F*(2, 41) = 0.10
Mean (SD)	(4.97)	(5.03)	(5.27)	*p* = 0.90

Baby weight in kg	3.7	3.6	3.4	*F*(2, 41) = 1.37
Mean (SD)	(0.75)	(0.43)	(0.38)	*p* = 0.27

Baby age in months	3.78	3.71	3.31	*F*(2, 42) = 1.40
Mean (SD)	(0.42)	(0.96)	(1.02)	*p* = 0.26

EPDS	4.8	12.8	15.6	*F*(2, 41) = 26.87
Mean (SD)	(3.40)	(4.85)	(4.65)	*p* < 0.0001

GAD-Q	1.5	8.6	7.2	*F*(2, 41) = 44.86
Mean (SD)	(1.53)	(2.38)	(2.44)	*p* < 0.0001

Mother educational background (% degree or higher)	40.0%	40.0%	40.0%	*χ*^2^(2) = 0.00 *p* = 1.0

Birth order (% primiparous)	33.3%	26.7%	28.6%	*χ*^2^(2) = 0.17 *p* = 0.92

**Table 2:** Participant emotional status effect on ratings of baby faces.

		Mean (SD)				Group comparison statistics		
		Total sample	Control	Anxiety	Depression	*F*	*p*	*η*^2^
Long presentation	Positive	6.5 (1.16)	7.00 (0.89)	6.00 (1.14)	6.6 (1.27)	2.85	0.07	.12
	Muted positive	5.00 (1.27)	5.4 (1.07)	4.5 (1.17)	4.9 (1.43)	2.24	0.12	.10
	Neutral	0.8 (1.50)	1.7 (1.49)	0.5 (1.41)	0.8 (1.64)	0.67	0.51	.03
	Muted negative	−3.6 (1.62)	−3.00 (1.70)	−3.6 (1.60)	−4.3 (1.38)	2.72	0.08	.11
	Negative	−5.8 (1.53)	−5.2 (1.89)	−5.6 (1.22)	−6.6 (1.05)	3.89	0.03	.16

Short presentation	Positive	6.0 (1.29)	6.2 (1.23)	5.7 (1.18)	6.2 (1.47)	0.78	0.47	.04
	Muted positive	4.5 (1.38)	4.9 (1.10)	4.3 (1.46)	4.1 (1.50)	1.38	0.26	.06
	Neutral	0.7 (1.42)	1.1 (1.39)	0.6 (1.19)	0.4 (1.65)	0.86	0.43	.04
	Muted negative	−2.6 (1.86)	−1.9 (1.84)	−2.5 (1.72)	−3.3 (1.88)	2.05	0.14	.09
	Negative	−4.2 (1.95)	−3.4 (2.04)	−4.3 (2.01)	−4.8 (1.61)	2.21	0.12	.09
